# Serotonin 5-HT_2A_ Receptor Activation Blocks TNF-α Mediated Inflammation *In Vivo*

**DOI:** 10.1371/journal.pone.0075426

**Published:** 2013-10-02

**Authors:** Felix Nau, Bangning Yu, David Martin, Charles D. Nichols

**Affiliations:** Department of Pharmacology and Experimental Therapeutics, Louisiana State University Health Sciences Center, New Orleans, Louisiana, United States of America; Max-Delbrück Center for Molecular Medicine (MDC), Germany

## Abstract

Tumor necrosis factor alpha (TNF-α) plays a key role in inflammation, and its production and signaling contribute to many inflammatory related diseases. Recently, we discovered that selective activation of serotonin 5-HT_2A_ receptors with the agonist (*R*)-DOI produces a super-potent blockade of proinflammatory markers in primary rat aortic smooth muscle cells. Here, we demonstrate that systemic administration of (*R*)-DOI can block the systemic effects of TNF-α in whole animal, with potent anti-inflammatory effects in the aortic arch and small intestine. This includes blockade of TNF-α-induced expression of pro-inflammatory cell adhesion (*Icam-1*, *Vcam-1*), cytokine (*Il-6, IL-1b*), and chemokine (*Mcp-1*, *Cx3cl1*) genes, and expression of VCAM-1 protein in the intestine. Further, systemic (*R*)-DOI also prevents the TNF-α-induced increase of circulating IL-6. Importantly, utilizing receptor selective antagonists, we have demonstrated that the mechanism underlying the systemic anti-inflammatory effects of (*R*)-DOI is activation of serotonin 5-HT_2A_ receptors. Our results highlight a powerful new role for the serotonin 5-HT_2A_ receptor in inflammatory processes, and indicate that agonism of serotonin receptors may represent an effective and novel approach to develop powerful small molecule therapeutics for inflammatory diseases and conditions such as atherosclerosis and inflammatory bowel disease.

## Introduction

Serotonin (5 hydroxytryptamine; 5-HT) is a neurotransmitter and hormone whose effects are mediated through interactions at seven different families of receptor proteins, comprised of 14 different subtypes, consisting of 13 G-protein coupled receptors and one ligand-gated ion channel [1]. Serotonin is primarily known for its function as a neurotransmitter within the CNS, and is involved in many processes including cognition and memory [2]. In the periphery, however, serotonin also plays significant roles where it mediates important processes like vasoconstriction and heart rate in the cardiovascular system [3], [4], and gastrointestinal function [5]. Although serotonin has been demonstrated to be involved in immune system function [6], its precise role remains unclear. Serotonin has been shown to influence a number of immunological processes, and can lead to both increases and decreases in proinflammatory cytokines [7], [8]. Significantly, individual serotonin receptors are known to be expressed in many immune-related tissues [9].

Recently, we discovered that (*R*)-1-(2,5-dimethoxy-4-iodophenyl)-2-aminopropane [(*R*)-DOI], a highly selective agonist at serotonin 5-HT_2_ receptors, super-potently inhibits tumor necrosis factor alpha (TNF-α) induced inflammation in primary rat aortic smooth muscle (RASM) cells [10]. The anti-inflammatory effects include inhibition of TNF-α-induced expression of adhesion molecules (*Icam-1*, *Vcam-1*), cytokines (*Il-6*), nitric oxide synthase activity, and activation and nuclear translocation of NFκB in RASM cells with an IC_50_ of ∼15 picomolar [10]. Significantly, we determined that the anti-inflammatory effects of (*R*)-DOI are exclusively mediated through activation of serotonin 5-HT_2A_ receptors [10].

Here, we have translated these findings to the whole animal, and show that systemic (*R*)-DOI has potent anti-inflammatory effects against the inflammation produced by systemic TNF-α in mice. The anti-inflammatory effects are most prominent in the aortic arch and small intestine. Importantly, we demonstrate that 5-HT_2A_ receptor activation is mediating the anti-inflammatory response.

## Materials and Methods

### Drugs

TNF-α was from Shenandoah Biotechnology (Warwick, PA). (*R*)-DOI was generously provided by Dr. David E. Nichols (Purdue University, IN). Each was dissolved in sterile physiological saline prior to use.

### Animals

Young adult male C57BL/6J mice were purchased from The Jackson Laboratory (Bar Harbor, ME, USA) and used for experiments in their 10^th^ week of age (mean weight of mice ± SEM on the day of treatment: 25.8±1.2 g). All animals were maintained in the animal care facility at LSUHSC in ventilated cages housed in a pathogen-free animal facility with free access to food and water. Animal protocols were prepared in accordance with the Guide for the Care and Use of Laboratory Animals and approved by the Institutional Animal Care and Use Committee at Louisiana State University Health Sciences Center.

### Induction of Systemic Inflammatory Responses Using TNF-α and Isolations for Systemic (*R*)-DOI and TNF-α

For the dose response experiments, mice were treated with sterile saline, TNF-α, and (*R*)-DOI as detailed: Group SS = Saline/Saline; Group ST = Saline/TNF-α (10 µg/kg); Group D3S = (*R*)-DOI (0.3 mg/kg)/Saline; Group D1T = (*R*)-DOI (0.01 µg/kg)/TNF-α (10 µg/kg); Group D2T = (*R*)-DOI (0.1 µg/kg)/TNF-α (10 µg/kg); and Group D3T = (*R*)-DOI (0.3 µg/kg)/TNF-α (10 µg/kg). Each mouse received intraperitoneal injections spaced 30 minutes apart, with saline or (*R*)-DOI first and TNF-α second. Five hours after the final treatment, mice were euthanized by a single injection of pentobarbital followed by cardiac puncture, exsanguination, and collection of blood. Blood was centrifuged in heparin coated tubes (BD, Billerica, MA) to isolate plasma, which was immediately stored at −80°C. Tissue samples were dissected and immediately frozen on dry ice and stored at −80°C until processing. The dose of TNF-α and timing of tissue harvesting was based on previous literature examination of TNF-α on proinflammatory markers in the mouse [11].

For the antagonist experiment, separate groups of mice were treated as follows: Group S = Vehicle/Saline/Saline; Group T = Vehicle/Saline/TNF-α (10 µg/kg); Group DT = Vehicle/(*R*)-DOI (0.3 mg/kg)/TNF-α (10 µg/kg); Group MDT = M100907 (1.0 mg/kg)/(*R*)-DOI (0.3 µg/kg)/TNF-α (10 µg/kg); Group MT = M100907 (1.0 mg/kg)/Saline/TNF-α (10 µg/kg); and Group M = M100907 (1.0 mg/kg)/Saline/Saline. Each mouse received intraperitoneal injections spaced 30 minutes apart, with M100907 or vehicle first, (*R*)-DOI or saline second, and TNF-α or saline third. Vehicle = 10% DMSO in sterile saline. Five hours after the final treatment, mice were euthanized by a single injection of pentobarbital followed by cardiac puncture. Blood and tissues were collected as described above.

### Tissue Sample Analysis: Cytokines and QPCR

**Figure 1:**
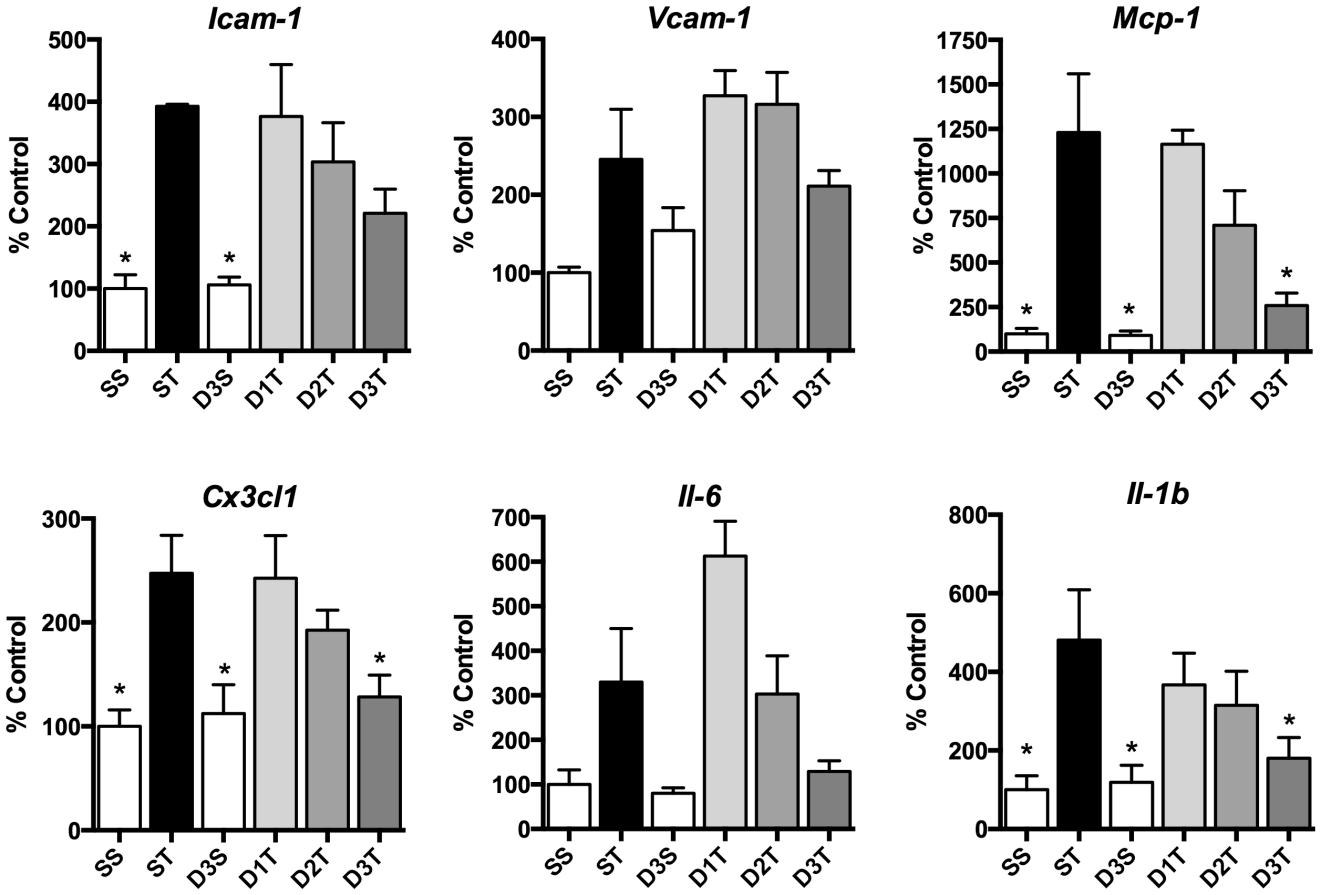
Systemic (*R*)-DOI inhibits TNF-α mediated pro-inflammatory marker gene expression in the aortic arch. There is blockade of inflammation that is significant at the 0.3/kg dose for *Mcp-1*, *Cx3cl1*, and *Il-1b* expression. Although not significant, there is a strong trend for anti-inflammatory effects on *Icam-1*, *Vcam-1* and *Il-6* expression. (SS = Saline/Saline control; ST = Saline/TNF-α; D3S = DOI (0.3 mg/kg)/saline; D1T = DOI (0.01 mg/kg)/TNF-α; D2T = DOI (0.1 mg/kg)/TNF-α; D3T = DOI (0.3 mg/kg)/TNF-α; * p<0.05 vs TNF-α; n = 4 animals per treatment; error bars represent ± SEM; ANOVA with Holm-Šídák post hoc test).

**Figure 2:**
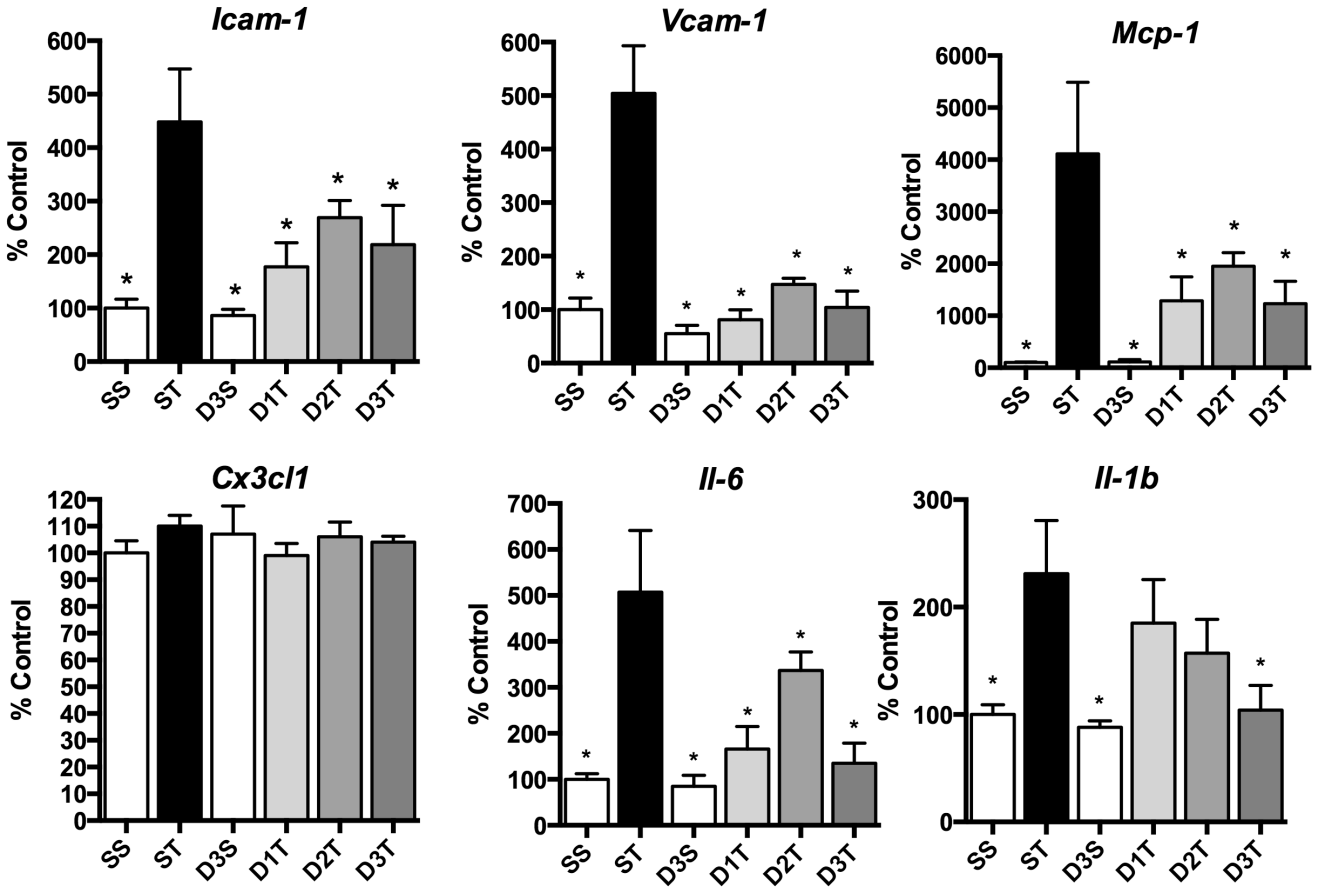
Systemic (*R*)-DOI inhibits TNF-α mediated pro-inflammatory marker gene expression in the small intestine. Even the lowest dose of 0.01/kg (*R*)-DOI can completely block inflammatory gene expression, indicating a super-potent effect in this tissue. There was no TNF-α-induced increase measured for *Cx3cl1* in this tissue. (SS = Saline/Saline control; ST = Saline/TNF-α; D3S = DOI (0.3 mg/kg)/saline; D1T = DOI (0.01 mg/kg)/TNF-α; D2T = DOI (0.1 mg/kg)/TNF-α; D3T = DOI (0.3 mg/kg)/TNF-α; * p<0.05 vs TNF-α; n = 4 animals per treatment; error bars represent ± SEM; ANOVA with Holm-Šídák post hoc test).

**Figure 3:**
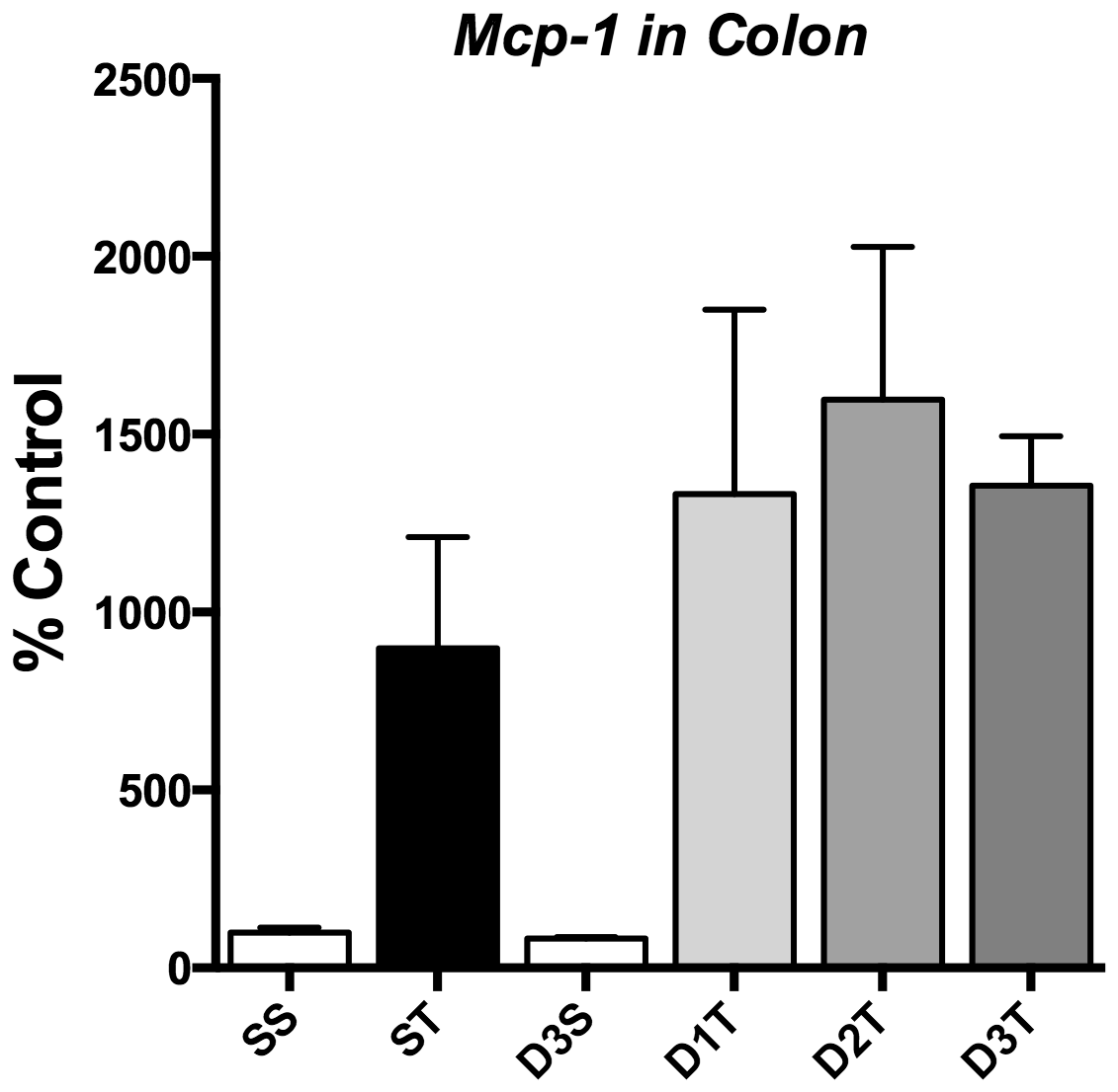
(*R*)-DOI has no effect on inflammatory gene expression in the colon. In contrast to the results observed in the aortic arch and the small intestine, (*R*)-DOI has no anti-inflammatory effects in the colon. Representative results for *Mcp-1* are shown here. (SS = Saline/Saline control; ST = Saline/TNF-α; D3S = DOI (0.3 mg/kg)/saline; D1T = DOI (0.01 mg/kg)/TNF-α; D2T = DOI (0.1 mg/kg)/TNF-α; D3T = DOI (0.3 mg/kg)/TNF-α; 4 animals per treatment; error bars represent ± SEM).

Cytokines in mouse blood plasma were assayed using the Milliplex Mouse Cytokine/Chemokine Panel kit (Millipore, Billerica, MA) following manufacturer’s directions on the Biorad Bioplex system (Hercules,CA). For all tissues, RNA was extracted using TRI Reagent-RT from Molecular Research Center, Inc. (Cincinnati, OH, USA) following protocols supplied by the manufacturer. First-strand cDNA was generated using the ImProm-II cDNA synthesis kit (Promega, Madison, WI, USA) following the manufacturer’s protocols with 500 ng total RNA per reaction. Quantitative real-time polymerase chain reaction (QPCR) was performed as described in QPCR Information S1. The sequences of primers used are as follows: *Icam-1*, forward 5′-CGAAGGTTCTTTTGCTCTGC-3′ and reverse 5′-GTCGAGCCGAGGACCATA-3′; *Vcam-1*, forward 5′- TGATTGGGAGAGACAAAGCA-3′ and reverse 5′- AACAACCGAATCCCCAACTT–3′; *Mcp-1*, forward 5′- GTGGGGGGTTAACTGCAT-3′ and reverse 5′- CAGGTCCCTGTCATGCTTCT-3′; *Cx3cl1*, forward 5′- CAGAAGCGTCTGTGCTGTGT-3′ and reverse 5′- CATCCGCTATCAGCTAAACCA -3′; *Il-1b,* forward 5′-AGCTGGATGCTCTCATCAGG-3′ and reverse 5′-AGTTGACGGACCCCAAAAG-3′; *Il-6*, forward 5′-GCTACCAAACTGGATATAATCAGGA-3′ and reverse 5′-CCAGGTAGCTATGGTACTCCAGAA-3′; *Htr2a*, forward 5′-TGATGTCACTTGCCATAGCTG-3′ and reverse 5′-AGAGCTTGCTGGGCAAAG-3′. Primers were synthesized by Integrated DNA Technologies, Inc (Coralville, IA, USA). Probe library probes from Roche Diagnostics (Universal Probe Library number) were as follows: U10, U83, U22, U80 U6, U38, and U3 for *Icam-1*, *Vcam-1*, *Mcp-1*, *Cx3cl1*, *Il-6*, *Il-1b*, and *Htr2a* respectively. Quantitative determination of gene expression levels using a two-step cycling protocol was performed on either a StepOne Plus (Applied Biosystems, Foster City, CA) to gather the data shown in Figures 1, 2 and 3, a MyIQ-5 Cycler (Bio-Rad, Hercules CA, USA) to gather the data shown in Figure 5, or a Roche LightCycler 480II LC (Roche, Indianapolis, IN) to gather the data shown in Figure 6. Relative gene expression levels were calculated using the 2[ΔΔC(T)] method. Levels of all targets from the test samples were normalized to internal *Gapdh* expression as determined using the Mouse Gapdh Gene Assay (Roche) in multiplex experiments. Additional information regarding sample preparation and QPCR protocols are provided in QPCR Information S1. Additional QPCR primer information is provided in Table S1.

### Western Blot Analysis

The organic phase of the TRI Reagent from the RNA isolations described above was collected and purified by dialysis using Slide-A-Lyzer G2 Dialysis Cassettes (Thermo Scientific) as described by Hummon et al. [12]. From the appropriate sample, 200 µl was added to dialysis cassettes and dialyzed against three changes of 0.1% Sodium Dodecyl Sulfate at at 4°C for 22 hours total. Dialysate was changed at 16 hrs, and 20 hours. The remaining product in the cassette was then subjected to further purification by precipitation in isopropanol as described in the TRI Reagent manufacturers protocol. The final purified protein pellet was dissolved in 100 µL of a 1∶1 mixture of 1.0% SDS and 8 M urea in 1.5 M Tris buffer (pH = 9.8). The proteins were quantified by BCA protein assay. From each sample, 76 µg of protein was loaded onto Mini-Protean TGX 4–20% precast gradient gels (BioRad) and ran for ∼1 hr at 120 V at room temperature. Gels were then transferred for 1 hour at 100 V onto Amersham Hybond-LFP membranes at 4°C (GE Healthcare). Membranes were blocked with 5% milk in 1×PBS (pH 7.4) for 1 hour and then probed overnight with primary antibodies to GAPDH (1∶1000) (Cell Signaling, #3683 S) and VCAM-1 (1∶200) (Santa Cruz, # sc-1504R) respectively. Blots were washed in 1×PBS (pH 7.4) with 0.05% Tween 20 (SIGMA) four times for 10 minutes each, and imaged on an ImageQuant LAS4000 gel imaging station (GE Healthcare) using SuperSignal West Pico Chemiluminescent Substrate (Thermo Scientific) following manufacturers directions. Quantitation was performed using ImageQuant TL software (GE Healthcare).

### Statistical Analysis

All statistical analysis was performed using GraphPad Prism (GraphPad, La Jolla, CA).

## Results

### (*R*)-DOI has Potent Anti-inflammatory Activity *in Vivo*

To explore the effects of systemic serotonin receptor activation on systemic TNF-α mediated inflammation *in vivo*, we pretreated normal young adult male mice with (*R*)-DOI (i.p.), followed 30 minutes later by treatment with a low dose of TNF-α (i.p.) that was predicted to produce a moderate systemic inflammatory response [11]. We examined the effects of three different doses of (*R*)-DOI: 0.01 mg/kg, 0.1 mg/kg, and 0.3 mg/kg. The highest dose we used, 0.3 mg/kg, is equal to the minimal dose needed to produce discrimination in the sensitive two-lever drug discrimination behavioral assay of 0.3 mg/kg [13]. Five hours after the TNF-α injection, the animals were sacrificed and various organs and tissues were removed for analysis. These included: brain, liver, aortic arch, adipose, small intestine, colon, kidney, and blood. Tissues were processed for RNA, and proinflammatory marker gene expression was analyzed by QPCR. Genes analyzed included cell adhesion markers (*Icam-1* and *Vcam-1*), cytokines (*Il-6; Il-1b*), and chemokines (*Mcp-1* and *Cx3cl1*).

We observed robust increases of inflammatory markers in many, but not all, of the tissues examined. Interestingly, we observed differential effects of (*R*)-DOI in different tissues. We saw no effect of (*R*)-DOI on TNF-α-mediated inflammation in the kidney, liver, or adipose tissues (data not shown). Consistent with our observations in cell culture with vascular cells, we observed a significant anti-inflammatory effect in the aortic arch for some markers where 0.3 mg/kg (*R*)-DOI (Figure 1) blocked TNF-α-induced expression of *Mcp-1, Cx3cl1,* and *IL-1b,* and produced a consistent trend for suppression of *Icam-1* and *Il-6* gene expression (Figure 1).

The anti-inflammatory effects measured in the small intestine were even more robust. Remarkably, we observed a near complete blockade of TNF-α-induced proinflammatory marker gene expression in this tissue at the lowest dose of (*R*)-DOI (0.01 mg/kg), mirroring the super potencies observed *in vitro* (Figure 2). The only gene that was not affected was *Cx3cl1*, where TNF-α did not appear to increase expression (Figure 2). There was a trend for a biphasic response to (*R*)-DOI in the intestine. Inverted U dose response curves for drugs at GPCRs are commonly observed. One possibility is that multiple pathways are being engaged at different drug levels that together contribute to the overall effect. Somewhat surprising, considering the results in the small intestine, was that no effects of (*R*)-DOI were observed in the colon (Figure 3).

**Figure 4:**
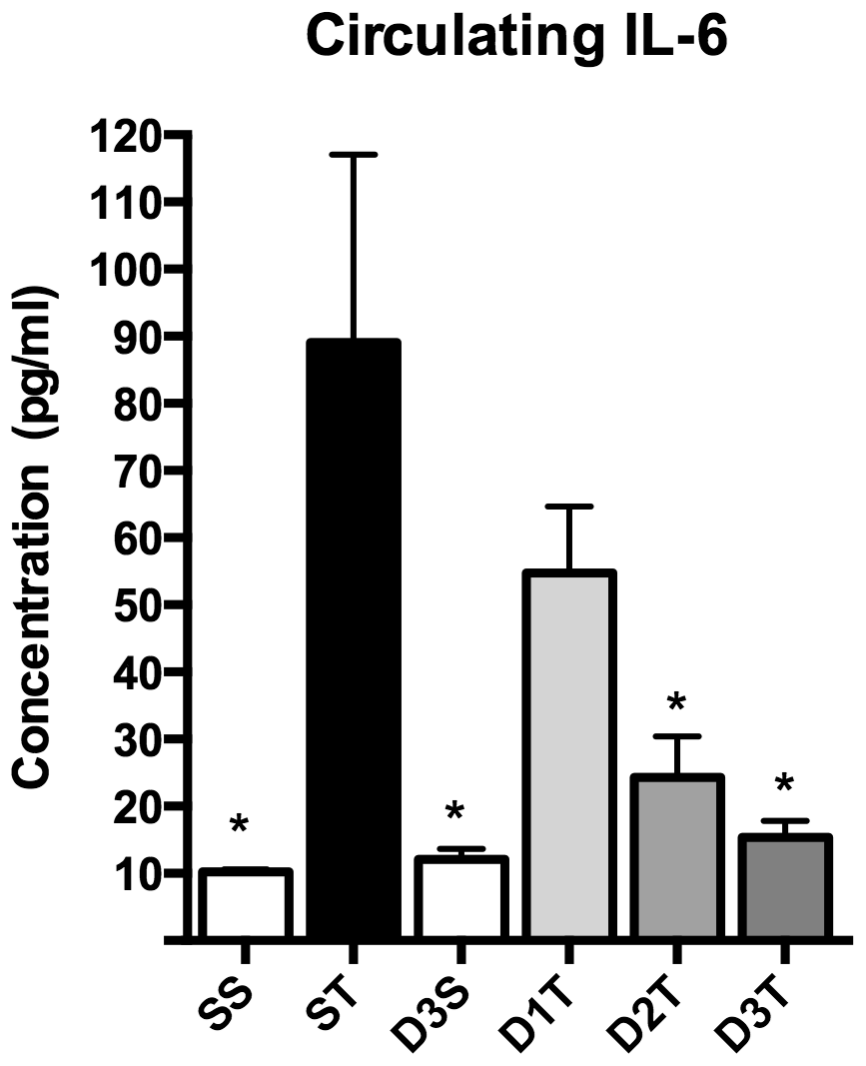
(*R*)-DOI blocks TNF-α induced increases in circulating IL-6. Systemic (*R*)-DOI prevented the TNF-α-induced increase of circulating IL-6 as determined by the Milliplex assay. (SS = Saline/Saline control; ST = Saline/TNF-α; D3S = DOI (0.3 mg/kg)/saline; D1T = DOI (0.01 mg/kg)/TNF-α; D2T = DOI (0.1 mg/kg)/TNF-α; D3T = DOI (0.3 mg/kg)/TNF-α; * p<0.05 vs TNF-α; n = 4 animals per treatment; error bars represent ± SEM; ANOVA with Holm-Šídák post hoc test).

To examine levels of circulating cytokines, we used the Milliplex Mouse Cytokine/Chemokine Panel, as described in the Methods section, to assess the levels of 32 circulating cytokines and chemokines at 5 hours post TNF-α. We found that five of these cytokines were significantly induced by TNF-α (not shown). Of these, the TNF-α-induced increase of IL-6 levels were significantly and completely blocked by the 0.1 and 0.3 mg/kg dose of (*R*)-DOI (Figure 4).

**Figure 5:**
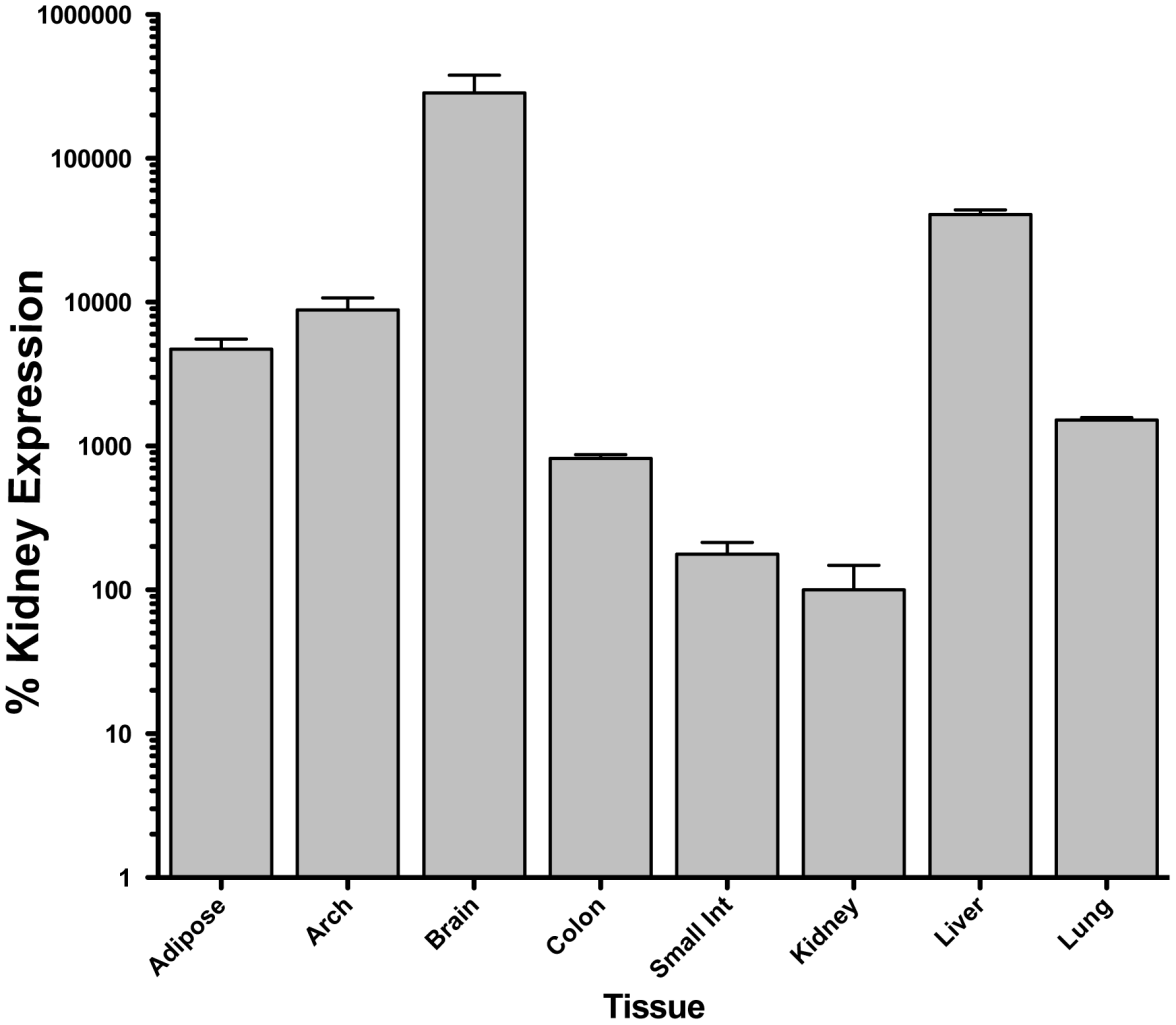
5-HT_2A_ receptor mRNA levels in different tissues. The relative levels of 5-HT_2A_ receptor mRNA in the mouse were determined by QPCR for various tissues examined in this study. Overall levels were, as expected, the highest in the frontal cortex of the brain (brain). The lowest levels observed were in the kidney. (Values on the Y-axis are presented in log-10 scale; all values are normalized to expression levels in the kidney, which were the lowest measured, and set to a value of 100% for comparison purposes only).

Because of the observed differential effects of (*R*)-DOI in the whole animal, we examined relative 5-HT_2A_ receptor expression in each of the tissues by QPCR. There appears to be no relationship between 5-HT_2A_ receptor mRNA expression and the ability of (*R*)-DOI to block the effects of TNF-α (Figure 5). For example, the brain has the highest expression of 5-HT_2A_ receptor mRNA, but there was no anti-inflammatory effect of (*R*)-DOI observed in brain.

### The Effects of (*R*)-DOI Against TNF-α-mediated Inflammation are Mediated Through 5-HT_2A_ Receptor Activation

**Figure 6:**
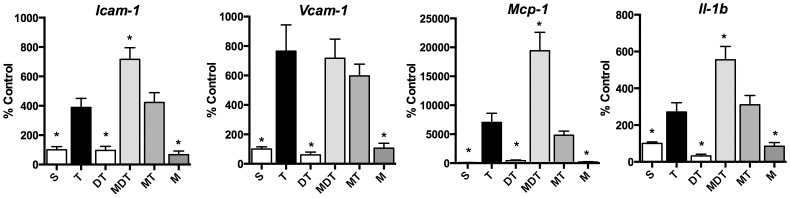
5-HT_2A_ receptor antagonist blocks the effects of (*R*)-DOI. Mice were pretreated with the selective 5-HT_2A_ receptor antagonist M100907 (1.0 mg/ml) (i.p.) 30 minutes prior to (*R*)-DOI (0.3 mg/ml; i.p.), followed 30 minutes later by TNF-α (i.p.). Pretreatment with antagonist blocked the effects of (*R*)-DOI to block TNF-α-induced pro-inflammatory gene expression as demonstrated here in the small intestine. (S = saline control; T = TNF; DT = DOI (0.3 mg/kg)+TNF; MDT = M100907 (1.0 mg/kg)+DOI (0.3 mg/kg)+TNF; MT = M100907 (1.0 mg/kg)+TNF; M = M100907 (1.0 mg/kg); n = 5 animals per treatment; * p<0.05 vs TNF-α; error bars represent ± SEM; ANOVA with Holm-Šídák post hoc test).

In our previous *in vitro* study, we used selective receptor antagonists to demonstrate that the anti-inflammatory effects of (*R*)-DOI were mediated through 5-HT_2A_ receptor activation. To address the primary mechanism underlying the anti-inflammatory effects of (*R*)-DOI in whole animal we pretreated mice with the 5-HT_2A_ receptor selective antagonist M100907 30 minutes prior to (*R*)-DOI. As is shown in Figure 6, administration of M100907 blocked the effects of (*R*)-DOI against TNF-α, indicating that the primary anti-inflammatory mechanism of (*R*)-DOI in whole animals is indeed through 5-HT_2A_ receptor activation.

### Treatment with (*R*)-DOI Blocks TNF-α Mediated Protein Expression in the Small Intestine

**Figure 7:**
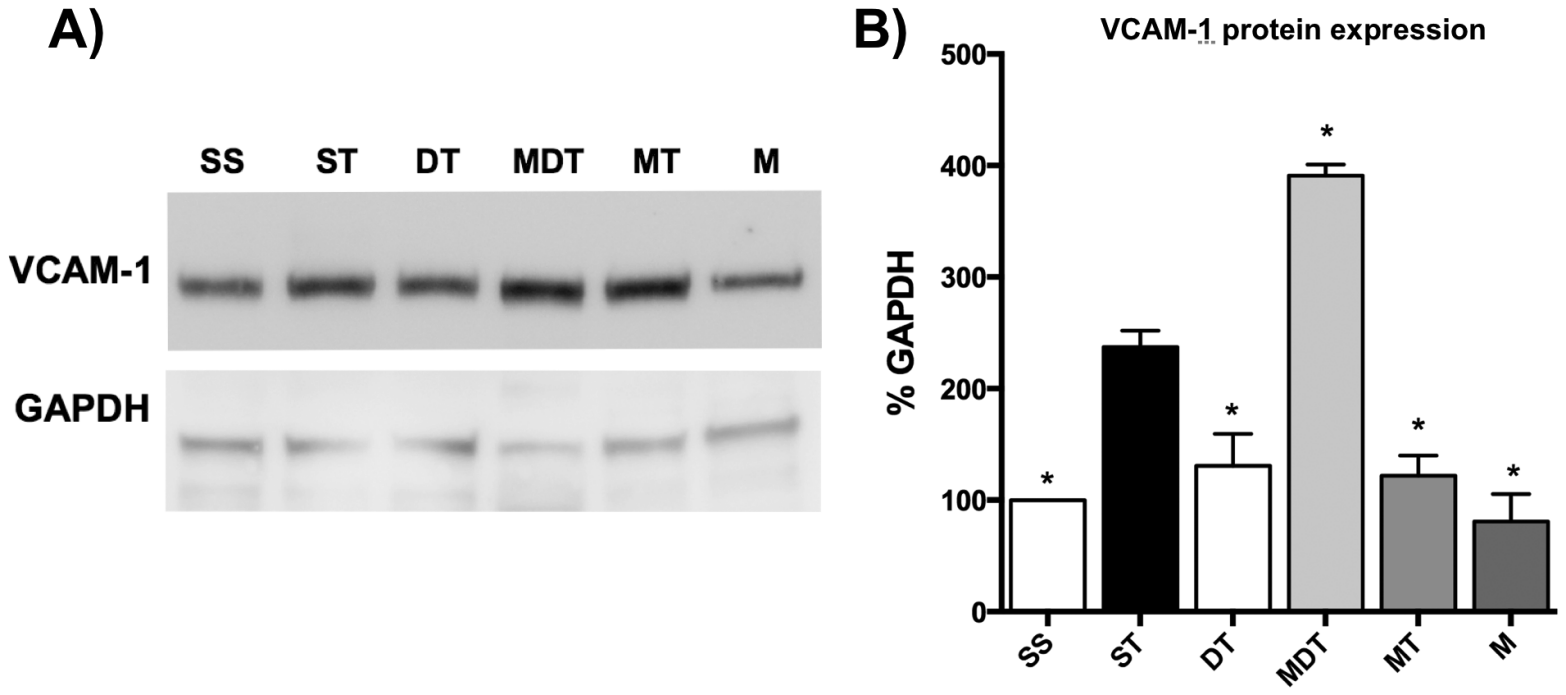
(R)-DOI blocks TNF-α mediated increases in Vcam-1 protein in the small intestine. A) Protein isolated from the same TRI-Reagent samples used to generate data shown in Figure 6 were western blotted and probed for Vcam-1 expression (representative blot shown). B) Results of quantitation of western blot. (*R*)-DOI blocks TNF-α mediated increases in VCAM-1 protein expression. Interestingly, M100907 also blocks this treatment, and the combination of (*R*)-DOI and M100907 potentiates the inflammatory response (S = Saline control; T = TNF; DT = DOI (0.3 mg/kg)+TNF; MDT = M100907 (1.0 mg/kg)+DOI (0.3 mg/kg)+TNF; MT = M100907 (1.0 mg/kg)+TNF; M = M100907 (1.0 mg/kg); n = 4 blots each with complete set of treatments corresponding to individual animals per treatment analyzed (n = 4 animals per treatment), with normalization between blots to the GAPDH band in the SS group for each blot; * p<0.05 vs TNF-α; error bars represent ± SEM; ANOVA with Holm-Šídák post hoc test).

To further confirm anti-inflammatory effects, we examined the ability of (*R*)-DOI to block TNF-α mediated increases in protein expression and measured Vcam-1 levels in the small intestine by western blot. We used tissue preparations from the same group of animals that were also treated with M100907 described above. The expression increase in Vcam-1 protein mediated by TNF-α was found to be more modest in comparison to increases in mRNA levels (2.5-fold vs. 7-fold) in the same set of animals. Nevertheless, pretreatment with (*R*)-DOI blocked TNF-α mediated increases in Vcam-1 levels (Figure 7). Interestingly, pretreatment with M100907 alone also blocked this increase at the protein level (Figure 7), but it does not at the mRNA level (Figure 6). The combination of both (*R*)-DOI and M100907 resulted in the potentiation of the TNF-α mediated increase in Vcam-1. These data raise the possibility that there are potentially multiple and interacting pathways modulating inflammation from the 5-HT_2A_ receptor.

## Discussion

We show here that the highly selective serotonin 5-HT_2_ receptor agonist (*R*)-DOI has potent anti-inflammatory effects in whole animal. These results are consistent with our previous *in vitro* study, where we reported that (*R*)-DOI super-potently blocks TNF-α mediated inflammation in primary aortic smooth muscle cells [10]. Importantly, we also show here that the primary mechanism for the anti-inflammatory effects of (*R*)-DOI is through activation of the 5-HT_2A_ receptor. We chose three different pretreatment doses of (*R*)-DOI to administer to mice to examine the dose response profile. Our highest dose of 0.3 mg/kg is the minimal necessary to produce any statistically significant behavioral effect in the highly sensitive two-lever drug discrimination assay [13]. This dose would be considered the threshold amount necessary for mice to detect a DOI-mediated interoceptive behavioral cue, and overall is considered by behavioral pharmacologists to be a low dose. For our intermediate anti-inflammatory dose, we chose 0.1 mg/kg, which although differs only slightly from the largest dose is not predicted to produce any rodent behavioral effects. As our lowest dose, we chose one an order of magnitude less, at 0.01 mg/kg. Our rationale was that the lowest dose was one that would likely be in the range to observe any super-potent effects, such as we had observed in our previous *in vitro* studies. The typical dose of DOI used for behavioral studies in mice is in the 1–3 mg/kg range [13]–[16]. The 10 µg/kg amount of TNF-α we used to induce inflammation in the mice is a dose that previous literature indicated would have a moderate and transient systemic inflammatory effect and induce proinflammatory gene expression in a variety of tissues, having a maximal effect at five hours post-treatment [11].

Analysis of the dissected tissues and collected blood revealed that some tissues did indeed respond to the anti-inflammatory effects of (*R*)-DOI. Consistent with our examination of aortic smooth muscle cells *in vitro*, we observed a trend that became significant at the 0.3 mg/kg dose for partial to complete blockade of proinflammatory marker gene expression induced by TNF-α in the aortic arch. The aortic arch has long been recognized as key vascular tissue affected by inflammation that ultimately leads to atherosclerosis [17]. Examination of blood plasma for a panel of circulating cytokines showed that there was a dose-dependent trend that became significant for blockade of TNF-α induced levels of IL-6, where the blockade was complete at 0.3 mg/kg (*R*)-DOI. It is not possible for us at this point to determine the site of action for blockade of IL-6 synthesis and release, but it is may be related to components of the vasculature, which are known to produce IL-6 like vascular smooth muscle cells. TNF-α mediated inflammation in vascular smooth muscle cells, as well as macrophages and endothelial cells, is believed to be a major mechanism underlying the pathophysiology of atherosclerosis [18], [19]. Additionally, 5-HT_2A_ receptors and TNF-α receptors are known to be expressed in immune related cells, and there may be a contribution from action at these cells contributing towards the overall observed effect of (*R*)-DOI. We show in Figure 1 that the highest dose of (*R*)-DOI (0.3 mg/kg) significantly and completely blocks TNF-α-induced *Mcp-1*, *IL-1b*, and *Cx3cl1* in the aortic arch. These correspond to proteins linked to the inflammation that leads to the development of atherosclerotic plaques [20]. For example, lack of IL-1b has been shown to reduce the severity of atherosclerotic plaques in ApoE deficient mice as well as reduce *Icam-1*, *Vcam-1*, and *Mcp-1* gene production [21]. Because TNF-α-induced expression of multiple pro-inflammatory genes is significantly blocked by (*R*)-DOI in whole aortic arch, 5-HT_2A_ receptor activation may be an effective approach to develop novel therapeutics for atherosclerosis. Importantly, therapeutic levels of this drug are far below those predicted to produce unwanted behavioral or other physiological effects.

In the small intestine we observed super-potent anti-inflammatory effects of (*R*)-DOI. The lowest dose of (*R*)-DOI, 0.01 mg/kg, consistently produced a maximal effect that was equivalent to the highest dose of drug, which for some markers meant complete blockade of the effects of TNF-α. Interestingly, no anti-inflammatory response to (*R*)-DOI was observed in the descending colon. Serotonin was identified in the gut before its role as a neurotransmitter was established, and has a long history of study in this tissue. Indeed, the vast majority of serotonin in the body is released from enterochromaffin cells within the intestinal mucosa of the gut, where it performs a number of functions [5], [22]. Serotonin is also synthesized and utilized in the enteric nervous system to regulate physiological processes [23]. Significantly, serotonin in the gut has been linked to inflammatory bowel disorders (reviewed in: [24]–[27]), although the exact nature of the role serotonin plays in these disorders remains largely unknown. There is some evidence that 5-HT_2A_ receptor function is involved in Irritable Bowel Syndrome, as it has been reported that certain gene polymorphisms are associated with an increased risk [28]. It is believed that inflammatory cytokines like TNF-α play a major role in the pathophysiology of inflammatory bowel diseases, and antibodies directed at either TNF-α or its receptor have proven to be effective therapies (reviewed in: [29]–[31]). Our results showing extremely potent blockade of multiple proinflammatory marker gene expression induced by TNF-α with (*R*)-DOI in the small intestine suggest that 5-HT_2A_ receptor agonism may be an effective novel small molecule therapeutic strategy to treat inflammatory bowel disorders.

Although we used a panel of five agonists at the 5-HT_2A_ receptor in our previous *in vitro* studies we only tested the most potent of them, (*R*)-DOI, here *in vivo*. Other agonists (e.g. LSD), or even 5-HT itself may have anti-inflammatory activity *in vivo* through activation of the 5-HT_2A_ receptor. As mentioned previously, the role of 5-HT in inflammation is not entirely clear. Serotonin can be either pro or anti-inflammatory depending on the tissue where it is acting. For example, in the bowel mucosa serotonin has pro-inflammatory effects whereas neuronal enteric serotonin has anti-inflammatory effects [32]. The exact 5-HT receptors mediating these effects, however, remain to be determined and our work may help to clarify the role of serotonin in certain types of inflammation.

Our results showing that blockade of 5-HT_2A_ receptors with the selective 5-HT_2A_ receptor antagonist M100907 has anti-inflammatory effects against TNF-α mediated Vcam-1 protein increases, but not *Vcam-1* mRNA increases suggest that there may be a separate *antagonist* mediated anti-inflammatory pathway that acts post-transcriptionally. There are reports that sarpogrelate, a 5-HT_2_ receptor antagonist, has anti-inflammatory properties [33]. There is also the possibility that M100907 is activating an effector pathway through functionally selective mechanisms that is contributing to its anti inflammatory effects. Interestingly, although M100907 alone did not affect TNF-α mediated increases in gene expression, gene expression of certain, but not all, inflammatory markers were increased significantly above TNF-α alone levels in the (*R*)-DOI+M100907+TNF-α group. That the combination of both (*R*)-DOI and M100907 can produce an enhancement of TNF-α mediated gene or protein expression further supports the notion of separate effector pathways, such that when the two treatments are combined it somehow produces an enhancement of the effects of TNF-α mediated inflammation. The nature of these potential interactions, however, remain to be elucidated in work beyond this study.

We also observed that the level of 5-HT_2A_ receptor mRNA expression detected in a particular tissue did not correlate with the anti-inflammatory effect of (*R*)-DOI. For example, although receptor expression in the aortic arch was nearly 10 fold higher than in the small intestine, the anti-inflammatory effects were found to be greater in the intestine. Further, we did not observe an anti-inflammatory effect in many tissues examined where receptor mRNA is expressed. These observations may indicate that anti-inflammatory pathway effectors are either more abundant or couple more efficiently to the receptor only in certain tissues like the small intestine to produce the desired anti-TNF-α effects through functionally selective mechanisms [34], [35]. For example, although we did not observe an anti-inflammatory effect of (*R*)-DOI in the liver, DOI has recently been shown to induce profound regeneration of the liver through activation of VEGF pathways [36]. This indicates that the effector pathways underlying regeneration and inflammation are likely different and represent functionally selective properties of signaling at this receptor such that the receptor does not activate anti-inflammatory pathways in the liver or that components of this pathway are absent in the liver and other non-responsive tissues. Another possible contributing factor to our observed lack of anti-inflammatory effects in certain tissues could be due to our method of administration. We injected (*R*)-DOI i.p., which is near the gut, and this may have had an influence on distribution to more distal tissues within 30 minutes. Further analysis with different routes of administration has indicated anti-inflammatory effects in additional tissues. For example, inhaled (*R*)-DOI has anti-inflammatory effects in the lung (unpublished data; manuscript under revision).

In summary, we have further characterized the anti-inflammatory effects of (*R*)-DOI activation of serotonin receptors and find potent anti TNF-α effects in whole animal. Blockade of systemic TNF-α-induced proinflammatory marker gene expression by (*R*)-DOI is most pronounced in the small intestine, and significant in the aortic arch. Furthermore, the TNF-α-induced increase in circulating IL-6, and expressed Vcam-1 protein in the small intestine are also entirely blocked by (*R*)-DOI. Importantly, using a 5-HT_2A_ receptor selective antagonist, we show that the primary mechanism for this anti-inflammatory activity is through activation of the serotonin 5-HT_2A_ receptor. Because we only used a 5-HT_2A_ receptor selective antagonist, it is not possible to rule out a role for the 5-HT_2B_ or 5-HT_2C_ receptors, but 5-HT_2A_ activity is both necessary and sufficient for the anti-inflammatory effects of (*R*)-DOI in whole animal. Further, there is a high density of 5-HT_2A_ receptors in the brain, not only in cortex [1] but also in many distinct brain nuclei that can mediate peripheral processes like sympathetic activity [37]. It is possible that (*R*)-DOI may have some action centrally that contributes to peripheral anti-inflammatory activity. Together, our results indicate that agonism of 5-HT_2A_ receptors is a novel approach to developing small-molecule based therapeutics for inflammatory diseases that involve TNF-α, especially for those of the vasculature and gut such as atherosclerosis and inflammatory bowel disease. Importantly, levels of drug needed to produce these beneficial effects are predicted to be up to orders of magnitude less than what are necessary to induce unwanted behavioral intoxication, and other physiological effects known to be influenced by 5-HT_2A_ receptor activity like vasoconstriction in the vasculature and gut function.

## Supporting Information

Table S1 **Summary table of QPCR primer information.**
(XLSX)

QPCR Information S1 **Information relevant to MIQE standards for QPCR.**
(DOCX)
