# Supplementary material for: Serotonin 5-HT2A Receptor Activation Blocks TNF-α Mediated Inflammation In Vivo
Source: PLoS One. 2013 Oct 2;8(10):e75426. doi: 10.1371/journal.pone.0075426 (PMC3788795; doi:10.1371/journal.pone.0075426)
Supplement: QPCR Information S1 — Information relevant to MIQE standards for QPCR. (DOCX) [file pone.0075426.s002.docx]

**SUPPLEMENTAL INFORMATION REGARDING QPCR EXPERIMENTS RELEVANT TO MIQE STANDARDS**

***Experimental Design***

Definition of Experimental and Control groups

Experimental Groups:

**Figure 1:**  ST, D1T, D2T, D3T

**Figure 2:** ST, D1T, D2T, D3T

**Figure 3:**  ST, D1T, D2T, D3T

**Figure 5:**  Multiple Tissues (adipose, aorta, brain, colon, small intestine, kidney, liver, lung)

**Figure 6:**  T, DT, MDT

Control Groups:

**Figure 1:** SS, D3S

**Figure 2:** SS, D3S

**Figure 3:** SS, D3S

**Figure 5:** All tissues normalized to Kidney Expression

**Figure 6:** S, MT, M

Number Within Each Group:

**Figure 1:** n=4

**Figure 2:** n=4

**Figure 3:** n=4

**Figure 5:** n=4

**Figure 6:**  n=5

Assays carried out by Dr. Yu (Figures 1,2, 3, 5) , and Mr. Nau (Figure 6), and not by core facilities.

***Tissue Sample Preparation***

*Description of Samples* (all from C57BL/6J mice; male; ~25 g)

All tissues were macrodissected.

**Figure 1:** ~2cm freshly dissected terminal ileum. (~2 cm from illeocecal valve)

**Figure 2:** Freshly dissected aortic arch (~0.5 cm long from aortic valve to first branch of aorta)

**Figure 3:** ~2cm freshly dissected colon. (~2 cm from illeocecal valve)

**Figure 5:** Freshly dissected tissue according to tissue name

**Figure 6:** ~2cm freshly dissected terminal ileum. (~2 cm from illeocecal valve)

*Mass of Samples Processed:*

**Figure 1:** Average weight of terminal ileum samples was ~100 mg

**Figure 2:** Average weight of aortic arch samples was ~30 mg

**Figure 3:** Average weight of colon samples ~125 mg

**Figure 6:** Average weight of terminal ileum samples was ~100 mg

*Processing Procedure:*

All tissues were promptly harvested after euthanasia and immediately frozen on dry ice in 1.5 ml tubes (tissues were frozen <1 minute after harvest). Once frozen, tissues were transferred to -80°C for 1-2 weeks to await nucleic acid processing.

*Sample Storage Conditions and Durations:*

All samples were stored at -80°C for at least 1-2 weeks before nucleic acids were extracted.

***Nucleic Acid Extraction***

Procedure: Samples were thawed in Tri Reagent RT and samples were hand homogenized in a 5 ml glass homoginizer.

Name of Kit: Tri Reagent RT nucleic acid extraction reagent and protocol provided (Cat# RT 111; Molecular Research Center, Inc., Cincinnati, OH)

Nucleic Acid Quantification: Absorbance at 260

Instrument and Method: Nanodrop ND-1000 Spectrophotometer and Nucleic Acid Quantification

Purity: Measured by absorbance ratio of A260/A230, RNA extracted from tissues had ratios at ~1.8.

Yield: On Average, 100 mg of tissue yielded 2,000 ng/μl of total RNA

***Reverse Transcription for 1^st^ Strand cDNA***

*Complete Reaction Conditions*: Comply completely with Section 4 of manual

Promega ImProm-II Reverse Transcription System (Catalogue #:A3800)

*Amount of RNA*: 500 ng RNA for all samples

Reaction Volume: 20 μL total for all cDNA reactions

*Priming Oligonucleotide and concentration:* Random Primers Catalogue #C1181, 0.5 μg/reaction

*Reverse Transcriptase and concentration:* 1 μL of ImProm-II Reverse Transcriptase Catalogue #: A3802 in 20 μL total reaction

*Temperature and time:* RNA tubes containing Random Primers (0.5 μg/reaction) and 500 ng of RNA were placed at 70°C for 5 minutes (5μL/reaction, normalized with nuclease free water) and then immediately placed on an ice bath for at least 5 minutes. Then, 15 μL of a separate experimental reaction (containing nuclease free water, ImProm-II 5x reaction buffer Part # M289A, MgCl_2_ 3mM Part # A351H, dNTP mix 0.5mM per each dNTP Catalogue # U1515, and ImProm-II Reverse Transcriptase) was added to the 5 μL RNA reaction from previous, on ice. This 20 μL tube was then placed in a MJ Research PTC-200 Peltier Thermal Cycler. Cycle Paramaters: annealing at 25°C for 5 minutes, extension for 42°C for 60 minutes, heat inactivation of ImProm-II Reverse Transcriptase with icubation at 70°C for 15 minutes.

*cDNA Storage Conditions*: cDNA reactions were stored frozen at -20°C until used in amplification reactions.

***qPCR Target Information and qPCR oligonucleotides***

Primer sequences, probe numbers from Roche universal LNA probe library (Product #04683633001) can be found in Supplementary Table 1. All primers and probe combinations were designed using the Roche Universal Probe Library Assay Design Center. These qPCR assays are designed with strenuous constraints by Roche to account for proper intron spanning requirments, GC content of primers, primer lengths, melting temperature of primers, amplicon lengths, and in silico PCR results. All assays were designed to be multiplexed with mouse GAPDH primer and Roche GAPDH probe assay (Roche Cat. no. 05046211001) and is listed in Supplementary Table 1. IDT manufactured all of the oligonucleotide primers, which were purified by standard desalting.

***qPCR protocol***

For data shown in Figures 1, 2, 4, and 5, USB HotStart-IT Probe Master Mix (Affymetrix; # 75766 500 RX) was used following manufactures instructions on the equipment as indicated below. For the data shown in Figure 6, Roche LightCycler 480 II Probe Master Mix was used. (Catalogue # 04887301001, Reference 04887301001, Version 9). cDNA was diluted at 1:5 ratio (4μL H2O and 1 μL cDNA). Internal control primers (GAPDH) were diluted 1:50 and primers for genes of interest were diluted 1:10. Total reaction volume was 20 μl/well and each reaction was run in triplicate. The concentrations of primers, probes, Mg^2+^ _,_ and dNTPs were in accordance with Roche LightCycler 480 Probes Master instruction manual (Catalogue # 04887301001, Reference 04887301001, Version 9). Negative controls containing no template cDNA were run with every PCR plate to check for contaminants.

*Manufacturer of Plates:*

USA Scientific 96 well white no skirt #1402-9589 (used with the Biorad iQ5)

Roche Light Cycler 480 Multiwell Plate 96, white (Ref 04729692001) (used

with the Light Cycler)

ABI 96 well MicroAmp Fast Optical #4346906 (used with the ABI StepOne Plus)

*Reaction setup*: Manual

*Manufacturer of qPCR Instruments to gather:*

Data shown in Figures 1, 2, 4: ABI StepOne Plus

Data shown in Figure 5: Biorad iQ5

Data shown in Figure 6: Roche Light Cycler 480 II

*Complete Thermocycling Parameters (used for all three qPCR Master Mixes and systems):*

Pre-Incubation (1 cycle) 95°C 10 min 4.4°C/sec Ramp Rate

Amplification (40 cycles) 95°C 10 sec 4.4°C/sec Ramp Rate

60°C 30 sec 2.2°C/sec Ramp Rate

72°C 1 sec 4.4°C/sec Ramp Rate

Cooling (1 cycle) 40°C 30 sec 2.2°C/sec Ramp Rate

***Data Analysis***

qPCR Analysis Program to determine C_q_ values for each sample:

ABI - StepOne™ System Software v2.1

BioRad iQ5 Optical System Software

Light Cycler 480 Software Release 1.5.0SP4 Version 1.5.0.39

Results for No template Controls (Negative Controls): Used 3 wells/mastermix batch with no template cDNA added, no amplification signal was observed for any of the no template controls.

Choice of Reference Gene: GAPDH

Description of Normalization Method: Relative gene expression levels were calculated using the 2[-ΔΔC(T)] method.

Number and stage of technical replicates: 3 technical replicates with 2 stage qPCR

Repeatability: The in-plate replicate variability was ~10% for all reactions.

Statistical methods for results significance: P-values noted on figures, for statistical significance p<0.05. ANOVA with post-hoc analysis was performed as described in the main text.

Statistical Software: Prism Version 5.0 and 6.0
